# Prediction of high-*T*_*c*_ superconductivity in heavy rare earth metals compressed Be-H alloy backbone

**DOI:** 10.1016/j.isci.2025.112098

**Published:** 2025-02-24

**Authors:** Yuxin Li, Chao Deng, Defang Duan, Hongwei Wang, Mingyang Du, Tian Cui

**Affiliations:** 1 Institute of High Pressure Physics, School of Physical Science and Technology, Ningbo University, Ningbo, China; 2 College of Physics, Jilin University, Changchun, China

**Keywords:** Superconductivity, Computational physics, Materials science

## Abstract

As the lightest element, hydrogen has the potential to become a room temperature superconductor upon metallization, though achieving this remains a significant challenge. Hydrogen-rich compounds leveraging the hydrogen pre-compression effect may offer promising alternatives for high-temperature superconductivity. In this study, we incorporated heavy rare earth elements into a fluorite-type Be-H alloy framework, resulting in the formation of XBeH_8_ (where X = Tm, Yb, Lu). This approach led to enhanced critical temperatures (*T*_*c*_) while maintaining stability at lower pressures. Specifically, TmBeH_8_ exhibits a *T*_*c*_ of 41–48 K at 80 GPa, whereas YbBeH_8_, which stabilizes at 100 GPa, demonstrates a *T*_*c*_ of 134–145 K. LuBeH_8_ achieves stabilization at 140 GPa and shows a remarkable *T*_*c*_ of 228–245 K. Despite LuBeH_8_ having a much higher *T*_*c*_ compared to LaBeH_8_, it shows lower stability at low pressures than LaBeH_8_. This research presents a viable pathway for designing high-*T*_*c*_ hydride superconductors under relatively moderate pressure conditions.

## Highlights

• Pre-compression of fluorite-type hydrogen alloy backbone using heavy rare earth atoms
• LuBeH_8_ achieves stabilization at 140 GPa with *T*_*c*_ of 228–245 K
• 4f electrons have a significant impact on the superconductivity of Be-H backbone

## Graphical abstract

**Figure:**
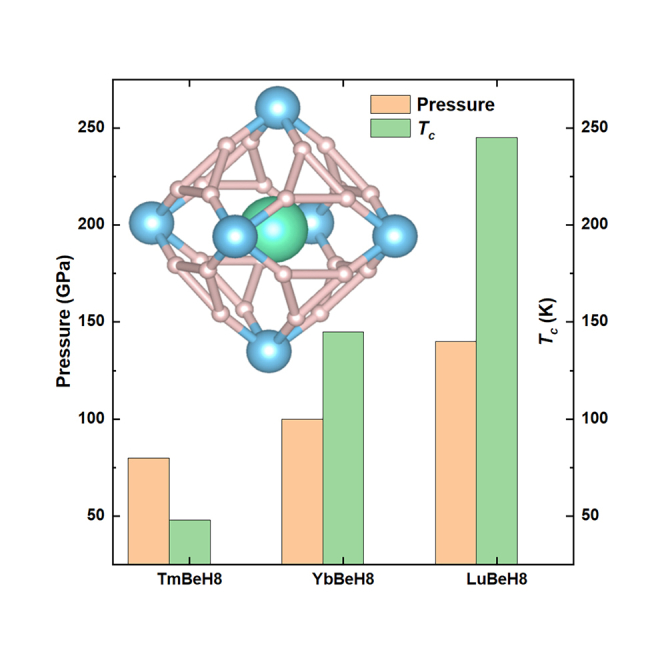

## Introduction

Since the discovery of mercury’s resistance-free conductivity at extremely low temperatures,^1^^,^^2^ the pursuit of room temperature superconductors has been a significant goal. It is found that hydrogen could metallize under high pressure emerged in 1935.^3^ By 1957, the BCS theory was introduced, establishing a relationship between the superconducting transition temperature (*T*_*c*_) and the Debye temperature.^4^ This theory positions hydrogen, the lightest element, as a potential candidate for room temperature superconductors post-metallization.^5^ Then, the challenge of hydrogen metallization has led to the proposition of pre-compression theory, which indicates that hydrogen-rich compounds may offer promising pathways to high *T*_*c*_ superconductors.^6^ The chemical pre-compression theory posits that incorporating other elements into synthesized superhydrides can be advantageous to reduce pressure required for stability than those required for pure hydrogen. Consequently, several significant superhydrides have been predicted to be promising superconductors with high *T*_*c*_ values,^7^^,^^8^^,^^9^such as CaH_6_,^10^ H_3_S,^11^ YH_6_,^12^ YH_9_,^13^ and LaH_10_,^13^^,^^14^ exhibited *T*_*c*_s exceeding 200 K, and these predictions were later experimentally verified.^15^^,^^16^^,^^17^^,^^18^^,^^19^^,^^20^^,^^21^

There has been significant interest in the exceptional superconductivity of clathrate hydrides XH_6_. Clathrate hexahydrides are prevalent among alkaline earth and rare earth metal superhydrides.^10^^,^^12^^,^^13^^,^^22^^,^^23^^,^^24^ These structures feature a body-centered cubic (bcc) arrangement with a metal atom at the center and H_24_ cages of hydrogen atoms filling the bcc lattice voids. CaH_6_ and YH_6_ have been experimentally synthesized, achieving high *T*_*c*_ s of 215 K at 172 GPa and 227 K at 166 GPa, respectively.^19^^,^^25^ Theoretically predicted *T*_*c*_ s for MgH_6_, ScH_6_, and LaH_6_ are 260 K at 300 GPa, 147 K at 285 GPa, and 174 K at 100 GPa, respectively.^13^^,^^22^^,^^23^ YbH_6_ and LuH_6_, both with fully filled *f*-orbitals, are forecasted to demonstrate high *T*_*c*_ superconductivity at relatively lower pressures (145 K at 70 GPa and 273 K at 100 GPa, respectively).^24^ TmH_6_, with partially filled 4*f* orbitals, can remain stable at 50 GPa but exhibits a comparatively low *T*_*c*_ of 25 K. Reports indicate that low-pressure stability in superhydrides can be maintained by *f* electrons, as seen in lanthanide clathrate hydrides like CeH_9_,^26^ PrH_9_,^27^ and NdH_9_.^28^ While filling the 4*f*-orbitals in metal atoms enhances structural stability at lower pressures, it may negatively impact superconductivity for fully filled atoms. The remarkable characteristics of TmH_6_, YbH_6_, and LuH_6_ highlight the potential of such structures for maintaining low-pressure stability.

In recent years, researchers have focused on predicting nearly all binary hydrides, leading to investigations into ternary hydrides formed by adding new elements to binary hydrides. Common structures in alkaline earth and rare earth metal hydrides include *Im*-3*m*-XH_6_, such as CaH_6_,^10^ MgH_6_,^22^ YH_6_,^12^^,^^13^^,^^14^ ScH_6_,^23^ and (Tm/Yb/Lu)H_6_.^24^ This structure can also extend into ternary forms like *Pm*-3*m*-ABH_12_, including (Y,Ca)H_6_,^29^^,^^30^^,^^31^, (Mg,Ca)H_6_,^32^ (Sc,Ca)H_6_,^33^ (La,Y)H_6_,^34^ and (Ca/Sc/Y,Yb/Lu)H_6_.^35^ Compounds such as CaH_6_^21^^,^^36^ and YH_6_^19^ have been successfully synthesized and show properties aligning with theoretical expectations. Notably, the cubic clathrate superhydrides La_x_Y_1-x_H_6_ have been synthesized experimentally through laser heating of yttrium-lanthanum alloys, demonstrating a *T*_*c*_ of 253 K at 183 GPa.^37^ This finding suggests that incorporating metal elements into clathrate hydrides can enhance compound stability.

Our group proposed a strategy for the rational design of high-temperature superconductors at low pressures.^38^ The famous clathrate backbone of LaH_10_ can be doped with a small-radius element X (X = Al, Be, B) to form an X-H alloy backbone, and then the ternary hydride AXH_8_ can be formed under the “chemical pre-compression” effect of the large-radius metal A (A = Ca, Sr, Ba, Sc, Y, La). The most outstanding one, LaBeH_8_, can even achieve high temperature superconductivity of 185 K at 20 GPa. Later, we found heavy metals Th and Ce can stabilize the X-H alloy backbone near ambient pressure. ThBeH_8_ was predicted to be stable at 7 GPa, with *T*_*c*_ of 113 K.^39^ Wan et al. predicted AcBeH_8_ can be stable at 50 GPa and exhibit the *T*_*c*_ of 203 K.^40^ Recently, LaBeH_8_ has been successfully synthesized in experiments and has been observed to have a *T*_*c*_ of 110 K at pressure of 80 GPa.^41^ Recently, we proposed high temperature superconductors with high stability by adding elements such as Yb/Lu on the basis of sodalite structure at medium pressure.^35^ Especially Y_3_LuH_24_ and YLu_3_H_24_, they exhibited a *T*_*c*_ of 283 K at 120 GPa and 288 K at 110 GPa, respectively. These results suggest that achieving room temperature superconductivity in hydride materials is feasible at moderate pressures. There have been studies estimating the superconductivity of LuBeH_8_, which have yielded quite astonishing results (355 K at 100 GPa). However, this work ignored the influence of *f* electrons on *T*_*c*_, which may lead to its results deviating significantly from the actual situation.^42^

The above work indicates that low-pressure stability in Be-H alloy backbone structure XBeH_8_ can also be maintained by *f* electrons. Also, partially filled 4*f*-orbitals may negatively impact superconductivity. Therefore, we selected Tm, Yb, and Lu elements to attempt to stabilize the Be-H alloy backbone at lower pressures. In this study, we explored the incorporation of heavy rare earth elements into a fluorite-type Be-H alloy framework, resulting in the formation of XBeH_8_ (where X = Tm, Yb, Lu) that demonstrates enhanced *T*_*c*_ while maintaining low-pressure stability. TmBeH_8_ remains stable at 80 GPa with a *T*_*c*_ of 41–48 K, whereas LuBeH_8_ achieves a *T*_*c*_ ranging from 228 to 245 K and requires stabilization at 140 GPa YbBeH_8_ shows moderate *T*_*c*_ and stabilization pressure, exhibiting a *T*_*c*_ of 134–145 K at 100 GPa. These findings provide valuable insights for the theoretical design of high-temperature superconductors.

## Results

### Crystal structure

**Figure 1:**
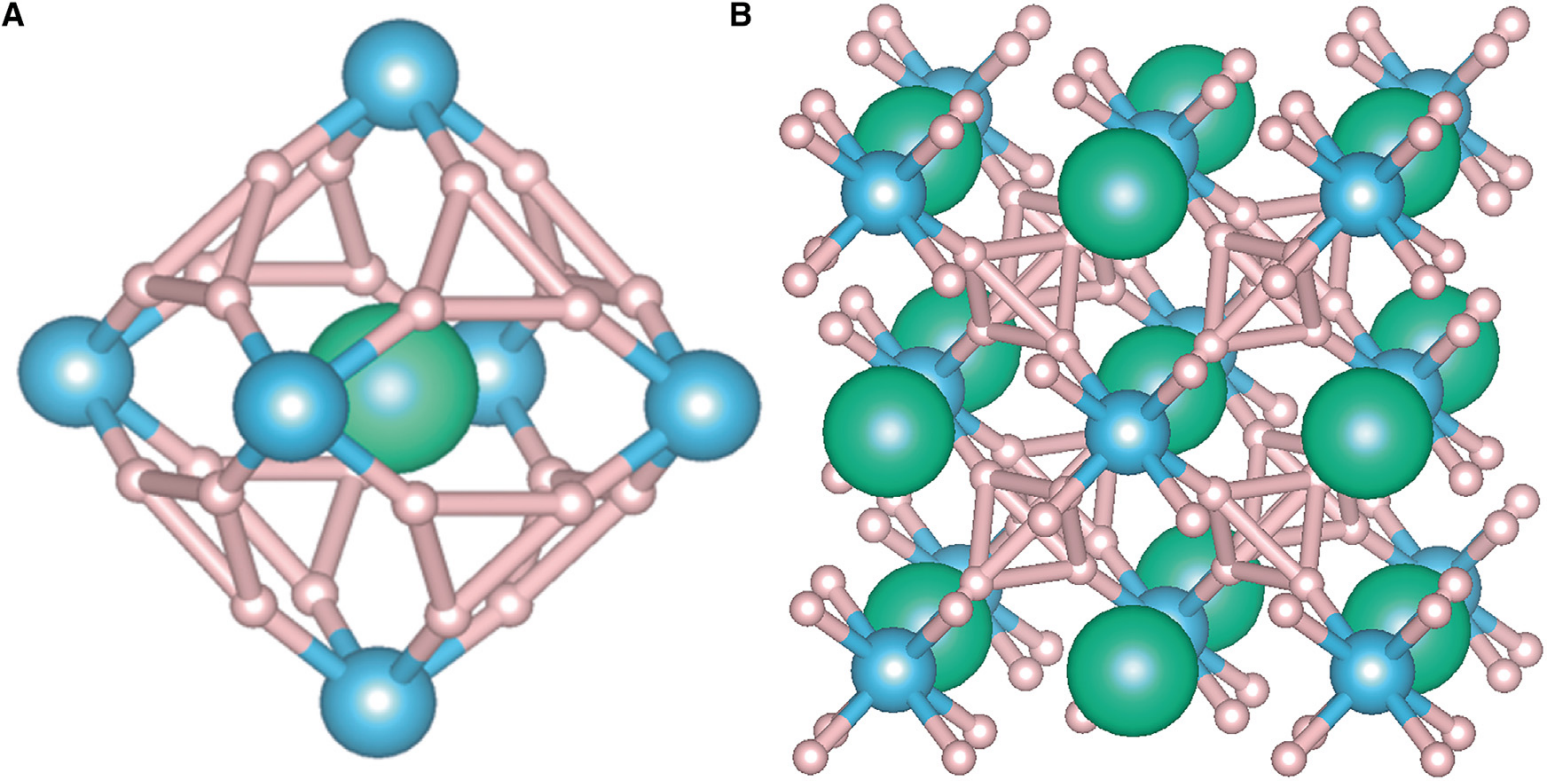
Structure features of XBeH_8_ (A) The fluorite-type cage of *Fm*-3*m*-XBeH_8_. (B) Crystal structure of Fm-3m-XBeH_8_. The green, blue, and pink spheres represent the metal atoms X (X = Tm, Yb, Lu), Be atoms, and H atoms, respectively.

First, we extend LaBeH_8_ to XBeH_8_, atom X is Tm, Yb, or Lu. As shown in Figure 1, green spheres are “pre-compressor” metal atoms X, blue spheres are Be atoms and pink spheres are H atoms, they occupy the 4*b* (0.5, 0.5, 0.5), 4*a* (0, 0, 0), and 32*f* (0.65526, 0.65526, 0.84474) Wyckoff positions, respectively. This kind of Be-H alloy backbone are arranged as fluorite-type structure, which is similar to LaH_10_, its H-H bond lengths are longer than common hydrides. This elongation is due to charge transferred from metal elements to the H-H bond and has a crucial effect on the low-pressure stability of this structure.

### Superconducting properties

**Figure 2:**
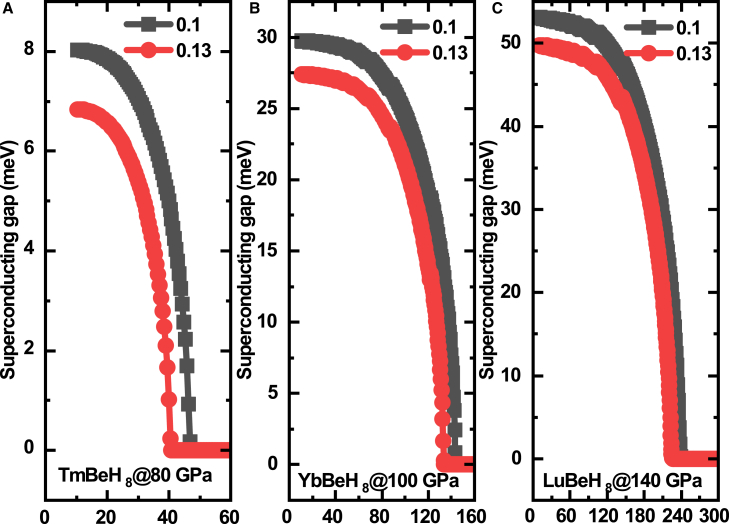
Superconducting properties of XBeH_8_ Calculated anisotropic superconducting gap of (A) TmBeH_8_ at 80 GPa, (B) YbBeH_8_ at 100 GPa, (C) LuBeH_8_ at 140 GPa. Coulomb pseudopotential μ∗ = 0.10 and 0.13.

The *T*_*c*_ s estimated through the self-consistent solution of the Eliashberg equation (scE)^43^ are shown in Figure 2. TmBeH_8_ has a *T*_*c*_ of 41–48 K at 80 GPa. Its electron-phonon coupling (EPC) parameter λ is 1.04, and logarithmic average phonon frequency ω_log_ is 657 K. This is not only higher than the minimum stabilization pressure of 50 GPa for TmH6, but also higher than the pressure of 20 GPa for LaBeH_8_. This means that Tm cannot stabilize the Be-H alloy backbone better than La, even after considering the effect of 4*f* electrons in enhancing the chemical precompression. Yb and Lu, which are also heavy rare earth elements adjacent to Tm. YbBeH_8_ can be stabilized at 100 GPa and exhibited *T*_*c*_ of 134–145 K. Its electron-phonon coupling (EPC) parameter λ is 2.41, and logarithmic average phonon frequency ω_log_ is 564 K YbBeH_8_ has a relatively high λ, but the *T*_*c*_ did not exceed 200 K. It only means that there are strong factors inhibiting its superconductivity, most likely from its 4*f* electrons. The relatively low ω_log_ of 564 K implies that its structure is on the verge of the boundary between stability and instability, and some softening is likely to occur on its phonon spectrum. LuBeH_8_ can be stabilized at 140 GPa with *T*_*c*_ of 228–245 K. Its electron-phonon coupling (EPC) parameter λ is 3.29, and logarithmic average phonon frequency ω_log_ is 642 K. Although the *T*_*c*_ of LuBeH_8_ is significantly higher than that of LaBeH_8_, it is far inferior to LaBeH_8_ in terms of low-pressure stability. For the Be-H alloy backbone, the central pre-compressed atoms should have sufficiently large radii, such as La, Ac, and Th. Our group^39^ and Gao et al.^44^ both proves this point.

### Stability

**Figure 3:**
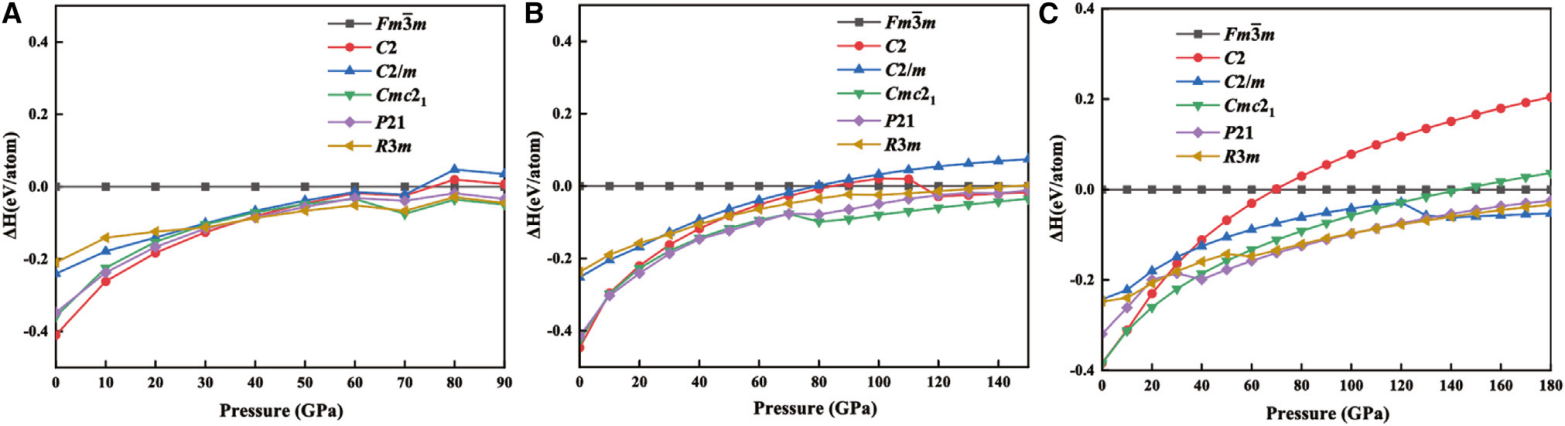
Enthalpies of TmBeH_8_ Calculated enthalpies per (A) TmBeH_8_, (B) YbBeH_8_, (C) LuBeH_8_ unit as the function of pressure.

To assess the thermodynamic stability of the *Fm*-3*m*-XBeH_8_ structures, we conducted structural searches at pressures ranging from 100 to 300 GPa, concentrating on XBeH_8_ compositions (where X = Tm, Yb, Lu) with 1–2 formula units. Our findings indicate that none of the *Fm*-3*m*-XBeH_8_ structures exhibit an enthalpy lower than that of the individual components, suggesting that they are all metastable phases (see Figure 3). Meanwhile, according to the constructed convex hull (see Figures S1–S3 in Supplementary Material), TmBeH_8_ is 38 meV/atom away from the convex hull, YbBeH_8_ is 71 meV/atom away from the convex hull, and LuBeH8 is147 meV/atom away from the convex hull. Consequently, synthesizing these structures experimentally will pose certain challenges. It is worth noting that metastable phases can be observed in experiments and may even outnumber thermodynamically stable phases.^45^

## Discussion

**Figure 4:**
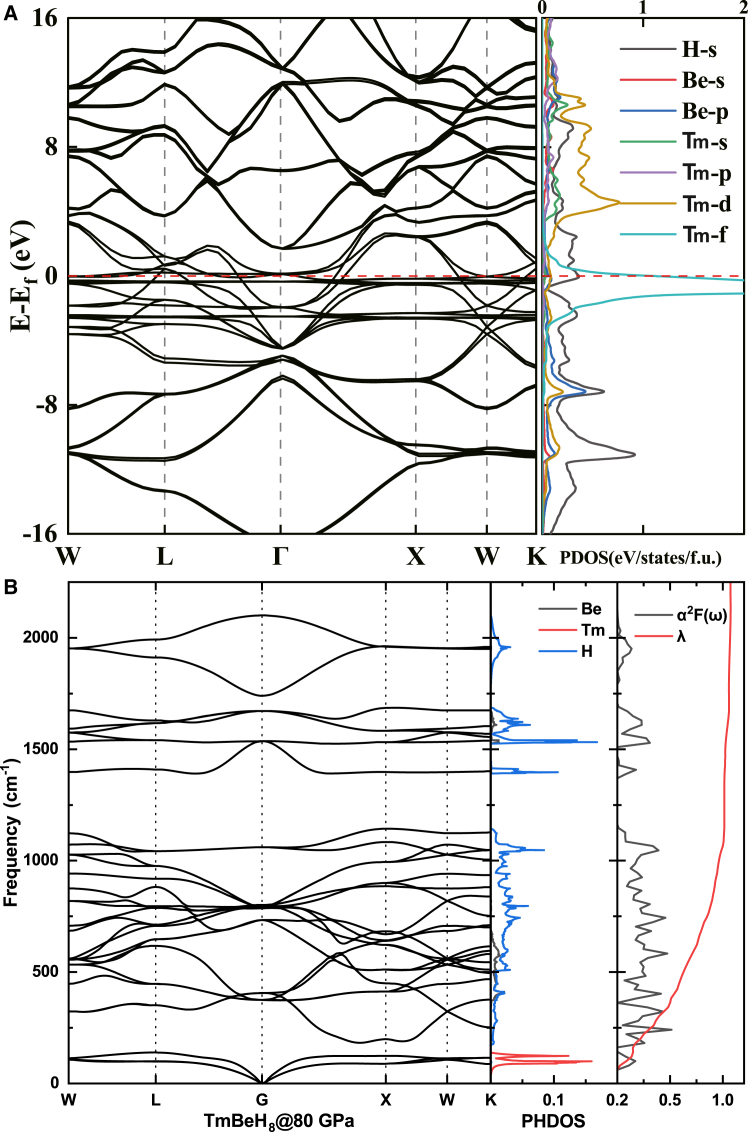
Band structure of TmBeH_8_ (A) The calculated electronic band structures and projected density of states for TmBeH_8_. (B) The calculated phonon band structure, PHDOS, electron-phonon coupling (EPC) parameter λ, and Eliashberg spectral function α^2^F(ω) of TmBeH_8_.

TmBeH_8_ displays a critical temperature of up to 48 K, which is likely influenced by the negative effects associated with Tm’s 4*f* electrons. To investigate this, we calculated the electronic band structures and projected density of states (PDOS). The electronic band structures reveal that the 4*f* orbitals from heavy rare earth elements create a series of localized, nearly non-dispersive bands (see Figure 4A). The positioning of these bands varies based on the outermost electrons of the element. For Tm, the bands linked to the partially filled 4*f* orbitals appear at the Fermi level. While these 4*f* electrons can enhance the chemical compression effects of metallic elements, aiding in structural stabilization at lower pressures, their proximity to the Fermi surface can negatively impact superconductivity. Specifically, the dominance of the 4*f* electrons at the Fermi level results in an extremely low density of states (DOS) for hydrogen. This low DOS is a key factor contributing to the reduced *T*_*c*_ of TmBeH_8_.

Superconductivity in superconductors primarily arises from strong electron-phonon coupling (EPC). To investigate this for TmBeH_8_, we calculated the phonon spectrum, projected phonon density of states (PHDOS), integral EPC parameter (λ), and the Eliashberg spectral function α^2^F(ω). By examining the frequency range where λ increases significantly, we can identify the vibrational modes critical to the superconductivity of this structure. As shown in Figure 4B, λ increases sharply in the 0–1200 cm^−1^ range, then rises more slowly between 1200 and 2100 cm^−1^. The total λ for TmBeH_8_ is 1.04, with a rapid increase to 1.01 in the 0–1200 cm^−1^ range, indicating that over 97% of the EPC arises from vibrational modes within this frequency range. Analyzing the PHDOS of the various elements allows us to pinpoint the sources of the vibrational modes in the 0-1200 cm^−1^ range. The rapid rise in λ is primarily attributed to the vibrations of Tm atoms in the 0-170 cm^−1^ range (indicated by red peaks in the PHDOS) and the vibrations of hydrogen atoms from 170 to 1200 cm^−1^ (shown by blue peaks in the PHDOS). Additionally, the phonon dispersion reveals soft phonon modes along the G-R-W direction, suggesting that the softening of the optical branch in the phonon spectrum significantly contributes to the electron-phonon coupling. This means that defect structures synthesized in future experiments may have higher superconducting transition temperatures. defects can play the dominant role in the high-temperature superconductivity, first-principles calculations indeed show that lattice vacancies can cause pressure-dependent phonon softening and substantially increase the electron-phonon coupling at high pressure.^46^ Sanna et al. predict that these defects effectively lower the vibrational frequency and increase the DOS at the Fermi energy, leading to a sizable increase of *T*_*c*_.

**Figure 5:**
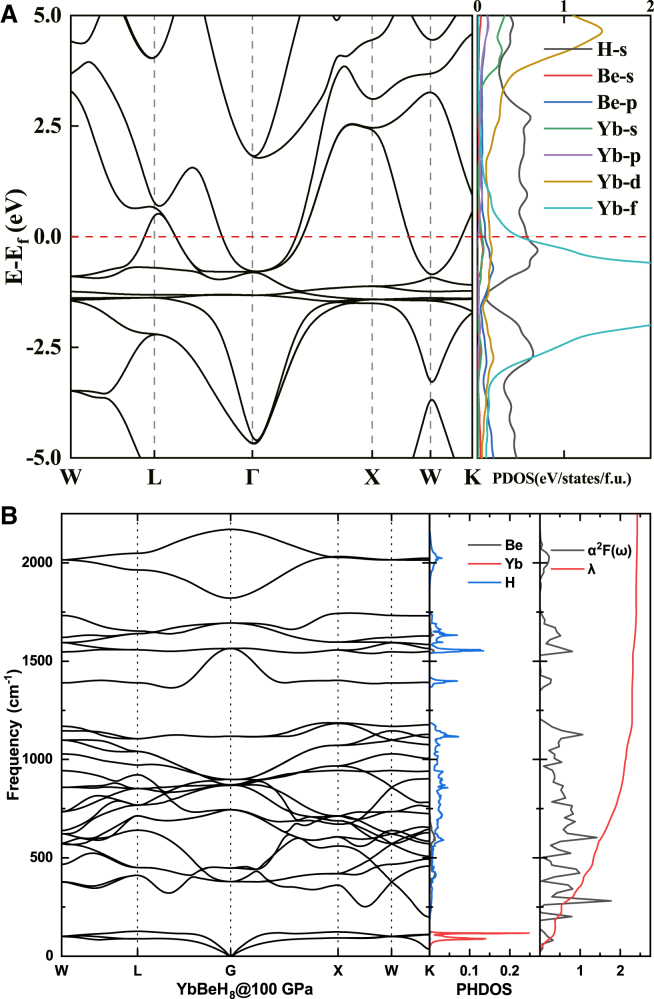
Band structure of YbBeH_8_ (A) The calculated electronic band structures and projected density of states for YbBeH_8_. (B) The calculated phonon band structure, PHDOS, electron-phonon coupling (EPC) parameter λ, and Eliashberg spectral function α^2^F(ω) of YbBeH_8_.

YbBeH_8_ exhibits a higher *T*_*c*_ of 134–145 K. The bands from the Yb atom, characterized by fully filled 4*f* orbitals, appear approximately 1 eV below the Fermi level (see Figure 5A). Similar to Tm, Yb also exhibits a strong chemical pre-compression effect but with fewer negative influences on superconductivity. The contribution of Yb’s 4*f* electrons at the Fermi surface is comparable to that of hydrogen’s s electrons. The calculated phonon spectrum, PHDOS, integral EPC parameter λ, and Eliashberg spectral function α^2^F(ω) for YbBeH_8_ are presented in Figure 5B. We observe that λ increases rapidly within the 0–1200 cm^−1^ range, then gradually in the 1200–2100 cm^−1^ range. The total λ for YbBeH_8_ is 2.41, with a swift rise to 2.295 in the 0–1200 cm^−1^ range, indicating that over 95% of the EPC comes from vibrational modes in this frequency range. By comparing the PHDOS of different elements, we find that the rapid increase in λ is primarily due to vibrations of Yb atoms in the 0–130 cm^−1^ range (red peaks in PHDOS) and hydrogen atoms in the 170–1200 cm^−1^ range (blue peaks in PHDOS). Furthermore, the phonon dispersion shows soft phonon modes near the K direction, with a key distinction from TmBeH_8_ being that the softening in YbBeH_8_ occurs in both the optical and acoustic branches.

**Figure 6:**
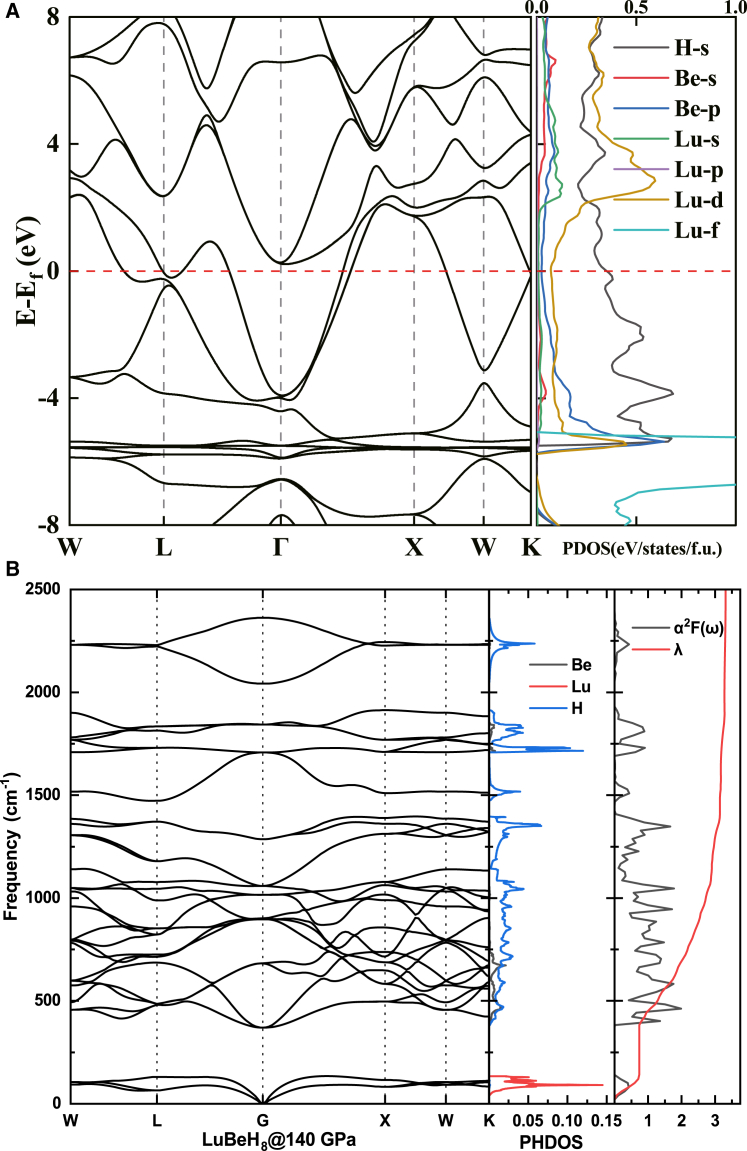
Band structure of LuBeH_8_ (A) The calculated electronic band structures and projected density of states for LuBeH8. (B) The calculated phonon band structure, PHDOS, electron-phonon coupling (EPC) parameter λ, and Eliashberg spectral function α^2^F(ω) of LuBeH_8_.

The bands associated with the Yb atom, which has fully filled 4*f* orbitals, are located approximately 1 eV below the Fermi level (refer to Figure 5A). In contrast, the bands from the Lu atom are situated about 6 eV below the Fermi level (see Figure 6A), a result of its fully filled 4*f* orbitals and an additional 5*d* electron. Consequently, the negative impact of Lu on superconductivity is minimized among heavy rare earth elements. Instead, the hydrogen electrons at the Fermi surface dominate, a characteristic common in high-temperature superconductors based on hydrides. LuBeH_8_ displays the highest *T*_*c*_ of 228–245 K among the three structures. The calculated phonon spectrum, projected phonon density of states (PHDOS), integral EPC parameter (λ), and the Eliashberg spectral function α^2^F(ω) for LuBeH_8_ are illustrated in Figure 6B. We observe that λ increases rapidly to 0.74 in the 0–160 cm^−1^ frequency range, remaining constant until the 340–2000 cm^−1^ range, where it rises sharply to 3.28, followed by a minimal increase. Compared to TmBeH_8_ and YbBeH_8_, the electron-phonon coupling in LuBeH_8_ is derived from a broader frequency range, with λ continuing to grow up to 2000 cm^−1^. The rapid rise in λ is primarily due to the vibrations of Lu atoms in the 0–160 cm^−1^ range (indicated by red peaks in the PHDOS) and the vibrations of hydrogen atoms in the 340–2000 cm^−1^ range (represented by blue peaks in the PHDOS). Notably, the contribution from hydrogen shows a significant increase, which is a key factor in the robust electron-phonon coupling observed in LuBeH_8_.

### Conclusions

In this study, we introduced heavy rare earth elements into a fluorite-type Be-H alloy backbone, allowing the newly formed XBeH_8_ structures (X = Tm, Yb, Lu) to achieve higher *T*_*c*_ values while maintaining stability at lower pressures. TmBeH_8_ exhibits a *T*_*c*_ of 41–48 K at 80 GPa, while Yb, also a heavy rare earth element adjacent to Tm, stabilizes at 100 GPa, showing a *T*_*c*_ of 134–145 K. LuBeH_8_, stabilized at 140 GPa, displays a *T*_*c*_ of 228–245 K. Although LuBeH_8_’s *T*_*c*_ is significantly greater than that of LaBeH_8_, it falls short of LaBeH_8_ in terms of low-pressure stability. For the Be-H alloy backbone, the central pre-compressed atoms must have sufficiently large radii. These findings provide an effective strategy for designing high-*T*_*c*_ hydride superconductors under relatively mild pressure conditions.

### Limitations of the study

Defects can play the dominant role in the high-temperature superconductivity. In this study, only ideal defect-free structures have been considered. We try to model a single vacancy in the high-pressure phases of LuBeH_8_ by removing an H atom from a supercell of the Fm-3m unit cell to calculate the effect of defects on the superconductivity of XBeH_8_. However, the heavy rare earth elements, Tm, Yb, and Lu contain *f* electrons, resulting in a considerable amount of computation. The supercell of XBeH_8_ has exceeded the limit that our node can compute.

## Resource availability

### Lead contact

Further information and requests for resources and reagents should be directed to and will be fulfilled by the lead contact, Mingyang Du (dumingyang@nbu.edu.cn).

### Materials availability

This study did not generate new unique reagents.

### Data and code availability

• The lead contact will provide access to the data presented in this study upon request.
• The lead contact will share the code used in this paper upon request.
• Any additional information required to reanalyze the data reported in this paper is available from the lead contact upon request.

## Acknowledgments

This work was supported by the 10.13039/501100001809National Natural Science Foundation of China (Grants No. 52072188, No. 12122405 and No. 12274169), Program for Science and Technology Innovation Team in Zhejiang (Grant No. 2021R01004), Jilin Provincial Science and Technology Development Project (20210509038RQ) and the Fundamental Research Funds for the Provincial Universities of Zhejiang (SJLY2023003). H.W. was supported by Yongjiang talents program 2024-4, the Foundation of Zhejiang Province Excellent Young Scientists, and the National Postdoctoral Program.

## Author contributions

Conceptualization and methodology, M.D.; investigation, M.D., Y.L., and C.D.; writing—original draft, M.D. and Y.L.; writing—review and editing, M.D. and Y.L.; funding acquisition, D.D., H.W., and T.C.; resources, M.D., D.D., H.W., and T.C.; supervision, M.D. and H.W.

## Declaration of interests

The authors declare no competing financial interest.

## STAR★Methods

### Key resources table

**Table:** 

REAGENT or RESOURCE	SOURCE	IDENTIFIER
**Software and algorithms**		

AIRSS	Materials Theory Group at University of Cambridge	www.mtg.msm.cam.ac.uk/Codes/AIRSS
CASTEP	Castep Developers Group	www.castep.org
Quantum-ESPRESSO	Quantum ESPRESSO project organization	www.quantum-espresso.org

### Method details

#### Structure search

The structural searches of XBeH_8_ (X = Tm, Yb, Lu) were performed from first principles using the AIRSS (Ab initio Random Structure Searching) code method,^47^^,^^48^ and the number of predicted structures at each XBeH_8_ stoichiometry is more than 1,000. Ab initio Random Structure Searching is a simple, and highly parallel, approach to structure prediction. The sensible random structures are constructed so that they have reasonable densities and atomic separations. They may embody crystallographic, chemical or prior experimental/computational knowledge. Beyond these explicit constraints the emphasis is on a broad, uniform, sampling of structure space. And then these structures are relaxed to nearby local energy minima using density functional theory (DFT) for the energies.

Structural relaxations during the structural searches are performed using the CASTEP (Cambridge Sequential Total Energy Package) code^49^ with ultrasoft pseudopotentials. The generalized gradient approximation^50^ was adopted within the Perdew-Burke-Ernzerhof framework^51^ for the exchange-correlation functional, and the projector augmented wave method^52^ was used to account for the interactions between electrons and ions. The cutoff energy was chosen to be 400 eV. A Monkhorst-Pack k-point mesh of 2π×0.07 Å^−1^ was used in preliminary structural relaxations during the structural searches.

#### Properties calculations

Final structural relaxations, enthalpy calculations, band structure analysis for XBeH_8_ were performed using the CASTEP code with ultrasoft pseudopotentials. The generalized gradient approximation^50^ was adopted within the Perdew-Burke-Ernzerhof framework^51^ for the exchange-correlation functional, and the projector augmented wave method^52^ was used to account for the interactions between electrons and ions. A cutoff energy of 1000 eV, and a Monkhorst-Pack k-point mesh of 2π×0.03 Å^−1^ were used, ensuring energy convergence with a precision of approximately 1 meV per atom (see Figures S5 and S6

In previous work, we evaluated the equation of state (EOS) for YbH_2_.^24^ Then it was compared with the experimental EOS to assess the reliability of our DFT calculations. For the low-pressure phase *Pnma* of YbH_2_, calculations using the on the fly pseudopotential poorly reproduce the experimental EOS; there is a significant volume discrepancy at the same pressure. The ultra-soft pseudopotential calculations substantially improve the agreement with the experiment; the agreement is further improved with the DFT+U scheme. One can see that there is a good agreement between the theory and experiment for the high-pressure phase *P*6_3_/mmc of YbH_2_, if we use the ultra-soft pseudo-potentials scheme. The authors of previous work concerned with ytterbium hydrides,^53^ used a Hubbard U = 5 eV for lower pressure phases and U = 0 eV for high-pressure phases to reproduce available experimental data, in clear agreement with our results.

#### Superconductivity calculations

Electron-phonon calculations of XBeH_8_ were calculated by the Quantum-ESPRESSO^54^ with projector-augmented-wave pseudopotential from VLab(http:/www.mineralscloud.com). Through density of state (DOS) and equation of state (EoS) comparisons, generated datasets were shown to yield excellent results comparable to highly accurate all-electron full-potential linearized augmented plane-wave plus local orbital (FLAPW+LO) calculations performed with the WIEN2K code.^55^ A cutoff energy of 90 Ry and a 24 × 24×24 k-point grid were used for Brillouin zone integration in electronic calculations (see Figures S7 and S8). And the EPC matrix elements were calculated in a 6 × 6×6 q-point grid. The valence electrons in the electronic states of Tm, Yb, and Lu atoms are 4f^13^5s^2^5p^6^6s^2^, 4f^14^5s^2^5p^6^6s^2^, 4f^14^5p^6^5d^1^6s^2^, respectively. The superconducting transition temperatures of these structures are estimated through the self-consistent solution of the Eliashberg equation (scE)^24^^,^^38^^,^^43^:

$$Z\left(i\omega_{n}\right)\Delta \left(i\omega_{n}\right)=\frac{\pi T}{N_{F}}\sum_{n^{\prime }}\frac{\Delta \left(i\omega_{n}^{\prime }\right)}{\sqrt{\omega_{n}^{\prime 2}+\Delta^{2}\left(i\omega_{n}^{\prime }\right)}}\times \left[\lambda \left(\omega_{n}-\omega_{n^{\prime }}\right)-N_{F}\mu^{*}\right]\delta \left(\epsilon \right)$$

$$\begin{array}{c} Z\left(i\omega_{n}\right)=1+\frac{\pi T}{N_{F}\omega_{n}}\sum_{n^{\prime }}\frac{\omega_{n}^{\prime }}{\sqrt{\omega_{n}^{\prime 2}+\Delta^{2}\left(i\omega_{n}^{\prime }\right)}}\lambda \left(\omega_{n}-\omega_{n^{\prime }}\right)\delta \left(\epsilon \right) \end{array}$$

where functions $Z\left(i\omega_{n}\right)$ and $\Delta \left(i\omega_{n}\right)$ are the renormalization function and pairing order parameter, respectively. $N_{F}$ is the density of electronic states at the Fermi level, and δ(ϵ) is the Dirac delta function. ${i\omega }_{n}=i\left(2n+1\right)\pi T_{c}$ are the fermion Matsubara frequencies. $\mu^{*}$ is the Coulomb pseudopotential, for which we use the widely accepted range of 0.1–0.13. $\lambda \left(\omega_{n}-\omega_{n^{\prime }}\right)$ contains the electron-phonon coupling matrix, phonon propagator, and the phonon density of states, and is given by:

$$\lambda \left(\omega_{n}-\omega_{n^{\prime }}\right)=\int_{0}^{\infty }d\omega \frac{2\omega }{{\left(\omega_{n}-\omega_{n}^{\prime }\right)}^{2}+\omega^{2}}\alpha^{2}F\left(\omega \right)$$

The equations for the $Z\left(i\omega_{n}\right)$ and $\Delta \left(i\omega_{n}\right)$ form a coupled nonlinear system and are solved self-consistently. We evaluated renormalization function and the order parameter for each Matsubara frequency along the imaginary energy axis. After calculating $Z\left(i\omega_{n}\right)$ and $\Delta \left(i\omega_{n}\right)$, an analytic continuation is performed to the real axis using Pade’ functions.

### Quantification and statistical analysis

Our study does not include statistical analysis or quantification.
