# Supplementary material for: Prediction of high-Tc superconductivity in heavy rare earth metals compressed Be-H alloy backbone
Source: iScience. 2025 Feb 24;28(3):112098. doi: 10.1016/j.isci.2025.112098 (PMC11928861; doi:10.1016/j.isci.2025.112098)
Supplement: Document S1. Figures S1–S8, Tables S1 and S2 [file mmc1.pdf]

iScience, Volume *28*

Supplemental information

Prediction of high-T_c superconductivity in heavy rare earth metals compressed Be-H alloy backbone

Yuxin Li, Chao Deng, Defang Duan, Hongwei Wang, Mingyang Du, and Tian Cui

FIGURES

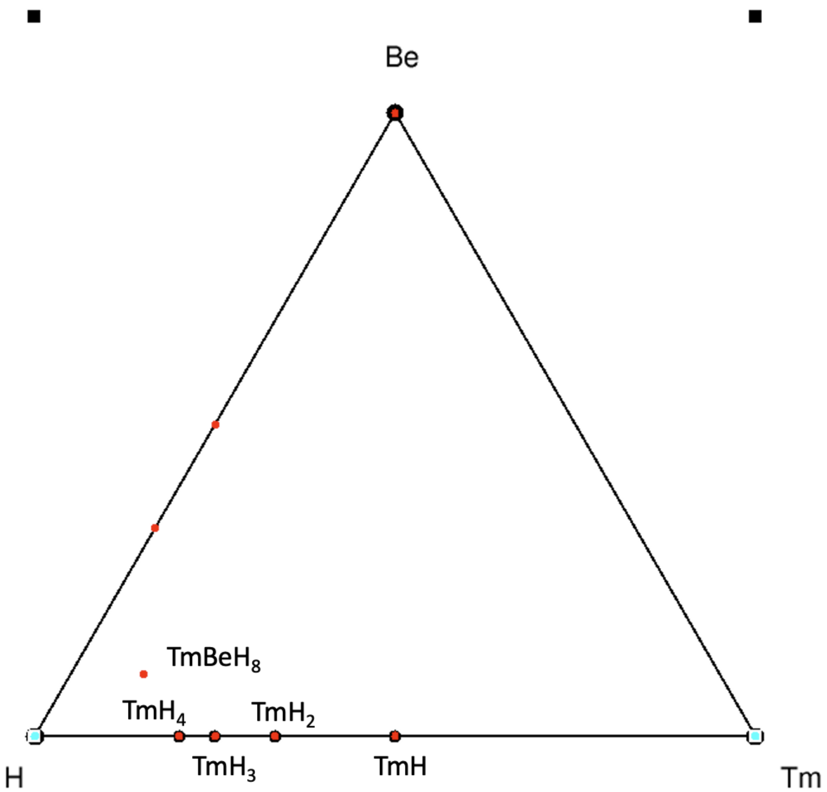

Figure S1 The convex hull of $TmBeH_8$ at 200 GPa.

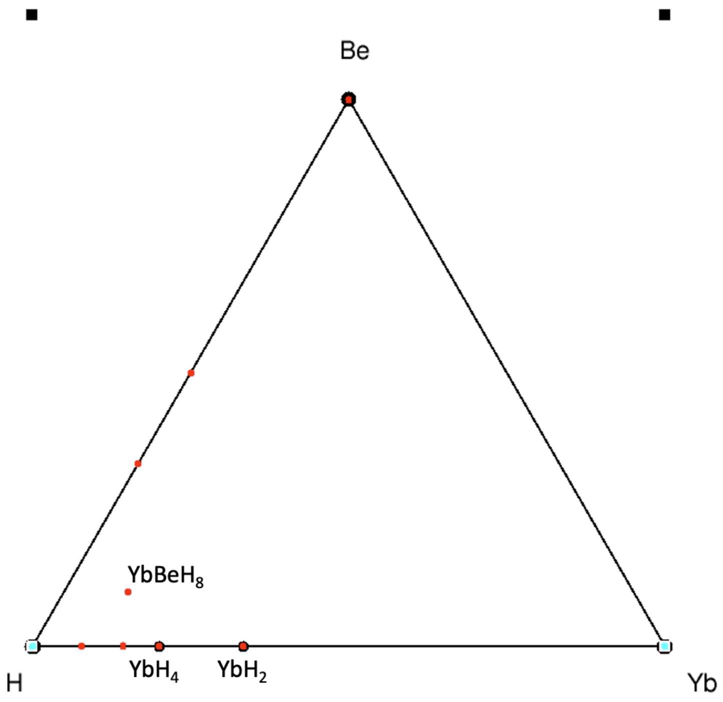

Figure S2 The convex hull of $YbBeH_8$ at 200 GPa.

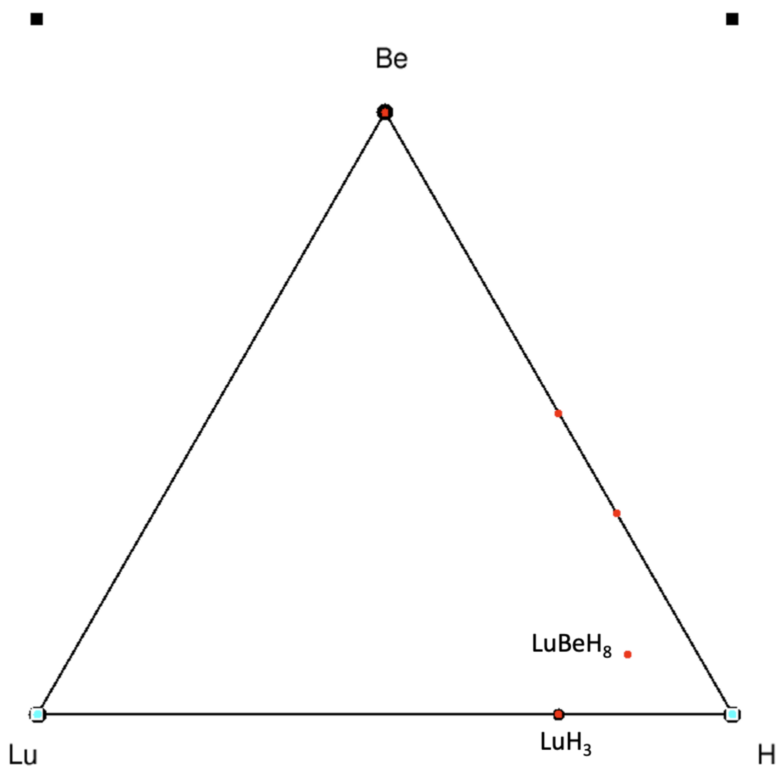

Figure S3 The convex hull of $LuBeH_8$ at 200 GPa.

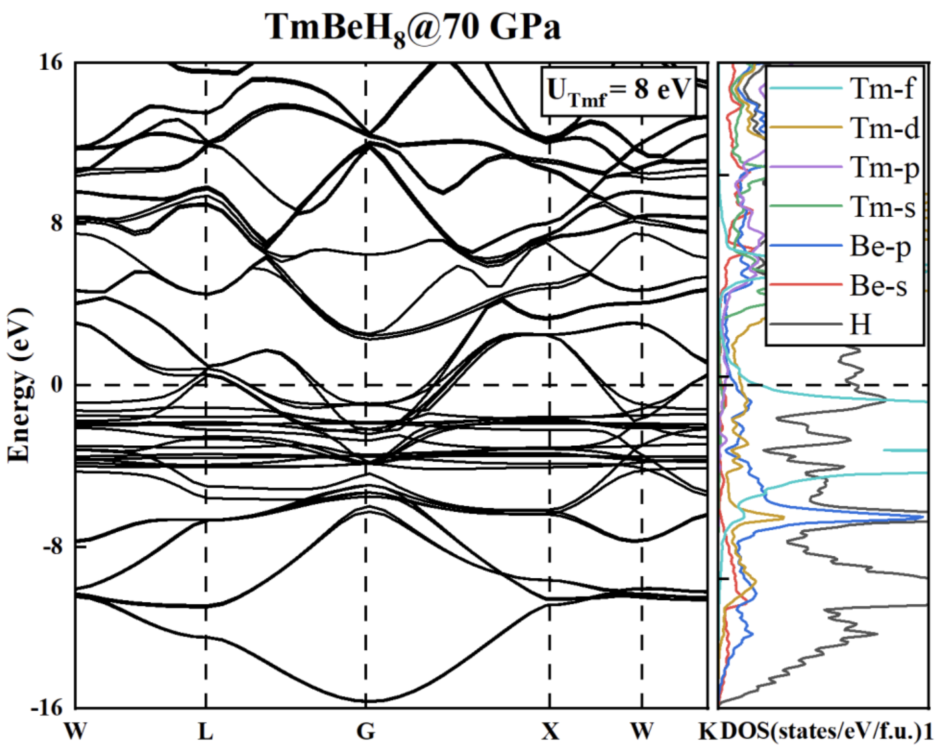

Figure. S4 Calculated electronic band structures and projected density of states for $TmBeH_8$ at 70 GPa with GGA+U (U= 8 eV).

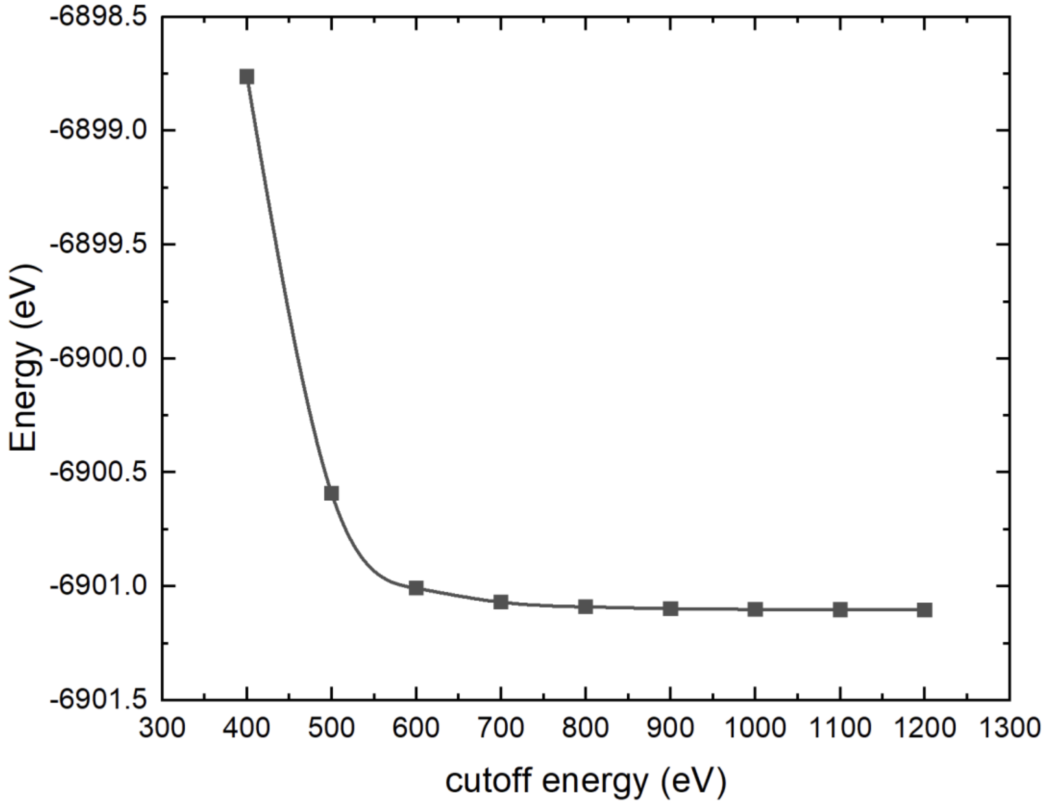

Figure. S5 The enthalpy of $YbBeH_8$ as a function of cutoff energy using CASTEP.

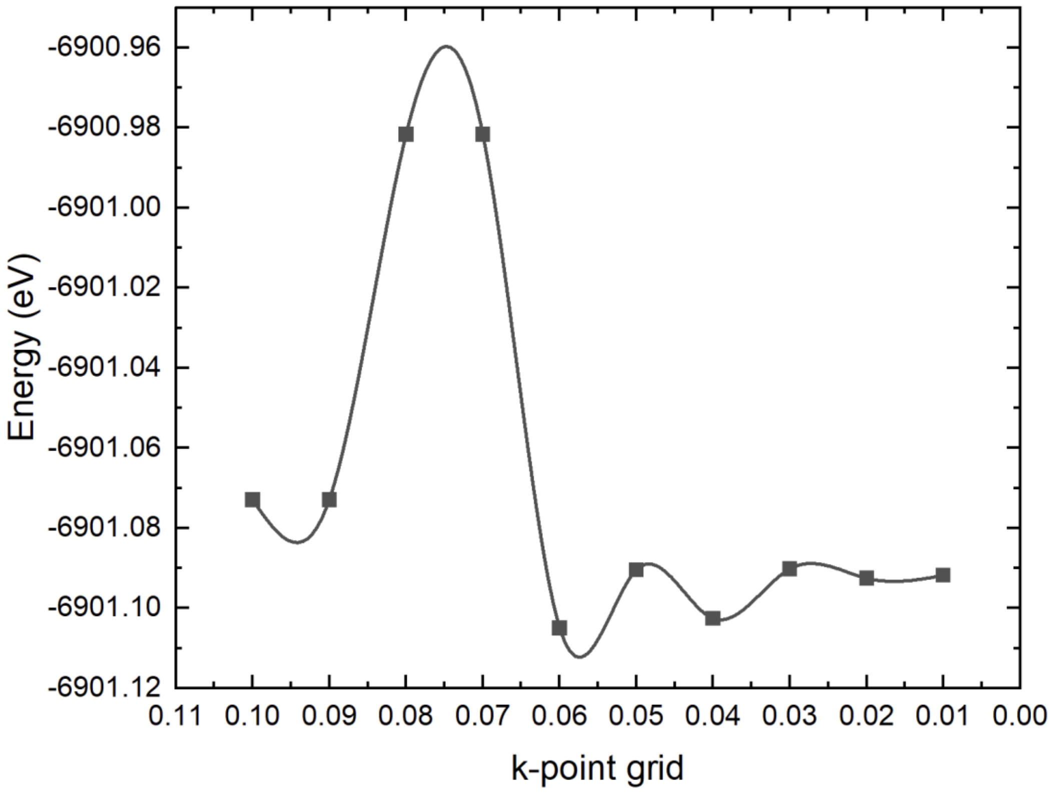

Figure. S6 The enthalpy of $YbBeH_8$ as a function of k-point grid using CASTEP.

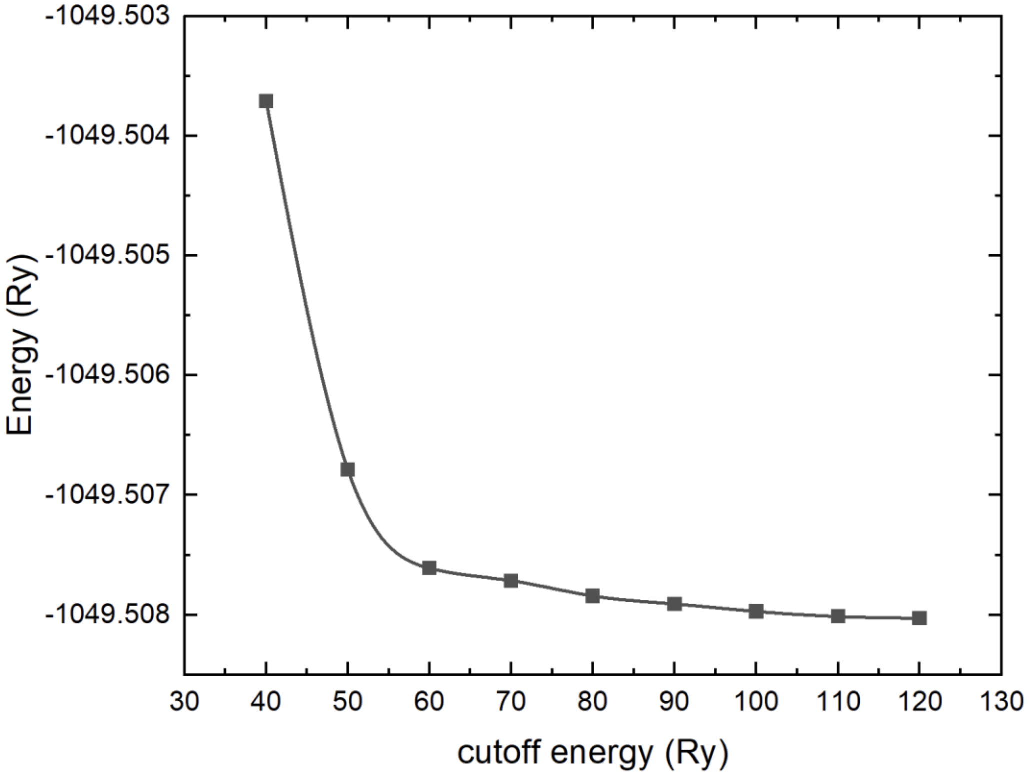

Figure. S7 The enthalpy of $YbBeH_8$ as a function of cutoff energy using QE.

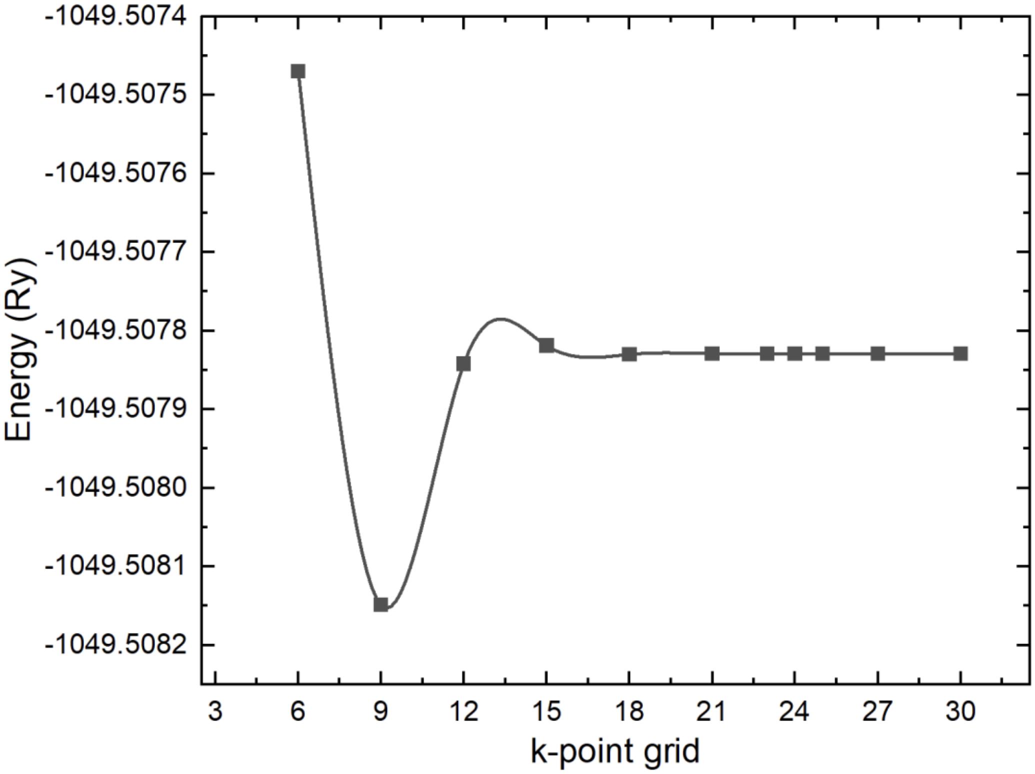

Figure. S8 The enthalpy of $YbBeH_8$ as a function of k-point grid y using QE.

TABLES

Table S1. The calculated electron-phonon coupling (EPC) parameter λ, logarithmic average phonon frequency ω_{log}, superconducting critical temperature $f_1 f_2 T_c$ using Allen-Dynes modified McMillan equation and T_c^{scE} using the Self-consistent solution of the Eliashberg equation for dynamically stable superconductors found in this work. The Coulomb pseudopotential $\mu^* = 0.10$ and 0.13.

Compound	Phase	Pressure (GPa)	λ	ω_{log} (K)	T_c^{scE} (K)
$TmBeH_8$	*Fm*-3*m*	80	1.04	657	41-48
$TmBeH_8$	*Fm*-3*m*	100	0.80	727	30-37
$TmBeH_8$	*Fm*-3*m*	150	0.65	917	21-27
$TmBeH_8$	*Fm*-3*m*	200	0.61	1028	18-25
$YbBeH_8$	*Fm*-3*m*	100	2.41	564	134-145
$YbBeH_8$	*Fm*-3*m*	150	1.48	909	121-134
$YbBeH_8$	*Fm*-3*m*	200	1.25	1041	110-124
$LuBeH_8$	*Fm*-3*m*	140	3.29	642	228-245
$LuBeH_8$	*Fm*-3*m*	200	2.35	835	215-230

Table S2. The calculated Sommerfeld constant γ, critical field $\mu_0 H_C(0)$, Δ(0) and R_Δ for dynamically stable superconductors found in this work.

Compound	Pressure (GPa)	γ , J/mol K^2	$\mu_0 H_C(0)$, T	Δ(0), meV	$R_\Delta = 2\Delta(0)/k_B T_c$
$TmBeH_8$	80	0.021	28.87	14.59	4.58
$YbBeH_8$	100	0.014	53.41	42.54	5.55
$LuBeH_8$	140	0.006	40.39	54.25	4.21
